# Modeling the significance of dynamic capability on the performance of microfinance institutions

**DOI:** 10.1371/journal.pone.0285814

**Published:** 2023-05-23

**Authors:** Hery Verianto The, Marvello Yang, Syed Ali Fazal, Maran, Jingzu Gao, Qing Yang, Abdullah Al Mamun

**Affiliations:** 1 Faculty of Economy and Business, Universitas Widya Dharma Pontianak, Pontianak, Kalimantan Barat, Indonesia; 2 Faculty of Entrepreneurship, Institute of Technology and Business Sabda Setia Pontianak, Pontianak, Kalimantan Barat, Indonesia; 3 UKM—Graduate School of Business, Universiti Kebangsaan Malaysia, Bangi, Selangor Darul Ehsan, Malaysia; Laval University, CANADA

## Abstract

According to strategic management theory, dynamic capability plays a significant role in enhancing organizational performance. Using a cross-sectional research design, the current study quantitatively assesses the mediating effect of dynamic capability on the relationships of total quality management, customer intellectual capital, and human resource management practice with the performance of microfinance institutions. An online survey involving 120 members of *Induk Koperasi Kredit*, a credit union association in West Kalimantan, Indonesia, is conducted. All the data are subjected to variance-based partial least squares structural equation modeling (PLS-SEM) analysis. The obtained results demonstrate the significant and positive influence of total quality management and human resource management practice on dynamic capability. Furthermore, dynamic capability is found to mediate the relationship between total quality management and human resource management practice on the performance of microfinance institutions. However, this study is unable to conclude that total quality management and human resource management practice have any significant impact on the performance of microfinance institutions. Nonetheless, this study demonstrates the crucial need for microfinance institutions to enhance their management activities via dynamic capability to enhance performance. This is one of the earliest studies conducted during the COVID-19 pandemic to examine the capabilities and performance of microfinance institutions in Indonesia. Notably, the performance of microfinance institutions can be further sustained by improving customers’ intellectual and dynamic capabilities.

## Introduction

Microfinance can effectively address market failures and help the poor and disadvantaged to provide solutions to their livelihood hardships, thereby making a significant contribution to the economy and society [1, 2]. The creation and existence of microfinance help the poor to access financial services that are essential for them, while facilitating smooth consumption and creating business opportunities for this group of people, thereby not only alleviating poverty in society but also increasing the participation of the poor in the formal economy as well as changing and promoting the economic development of a country [2–4]. Microfinance institutions (MFIs) can provide services and products such as microcredit, savings schemes, financial payment facilitation, micro-pension and micro-insurance schemes, and remittance facilitation to poor and vulnerable people in response to policy and institutional imperfections in credit, banking, and insurance markets [5]. Notably, MFIs provide poor people with access to financial services and support, which in turn help them maintain financial sustainability [6]. In a dynamic marketplace, MFIs must be able to integrate, expand, and reconfigure their internal and external capacities; manage and maintain their perspective forms; and respond quickly to business changes [7]. However, while MFIs are currently economically functional in running microfinance, maintaining or improving their performance under the influence of competitive external market environment factors and internal organizational factors can still be challenging [2, 8].

Based on the purpose and specificity of the operation of MFIs, masses in countries and regions with high poverty rates are more inclined to opt for microfinance, which is common in countries in the Asian region, such as Indonesia, India, and Pakistan [9, 10]. According to United Nations High Commissioner for Refugees [11], refugees and low-income households in Indonesia can receive a total of approximately US$ 12.5 million per year from MFIs, including financial and social services [12]. The assets and membership of MFIs in Indonesia have expanded dramatically from 356,327 in 2000 to 3,636,559 in 2020 [13]. Owing to the high demand for microfinance in Indonesia, MFIs are highly adaptable therein, thereby facilitating strategic measures to gain competitive advantages and sustain their performances [14]. However, this also typifies that the performance measures of MFIs are considered more comprehensive than those of traditional banks and credit institutions, which in turn leads to the need for a holistic approach to the assessment and enhancement of MFIs’ performance. Therefore, this study examines the factors that contribute to the performance of MFIs from three perspectives, namely, organization, employee, and customer, and explores their mediating role and impact on the performance and sustainability of MFIs through the dynamic capabilities of the organization.

The resource-based view (RBV) framework argues that organizations can gain sustainable competitive advantages through unique resources and firm-specific capabilities to maintain performance in this rapidly evolving environment [15]. Owing to the unique resources (customer base, funding sources etc.) of MFIs and their operational and business models, distinguishing them from traditional banks and credit institutions, this study employs the RBV to shed light on how MFIs respond and adapt to changes in dynamic business and market environments and provides valuable insights into the various organizational practices of MFIs to gain sustainable competitive advantage [16]. Previous studies have discussed the impact of total quality management (TQM), customers’ intellectual capital (CI), and human resource management practices (HRP) on organizational performance from both organizational and customer perspectives [17, 18]. There is also a strong correlation between an organization’s resources and capabilities and its performance as well as the development and enhancement of dynamic capabilities by purposefully creating, extending, or modifying their performance or service offerings, which in turn helps the organization to better adapt to dynamic market changes [19].

However, for countries such as Indonesia, which has a large number of low-income people, the determinants of organizational performance are related to the sustainability of the organization and enhanced returns, especially for financial institutions such as MFIs, where organizational performance is related to the survival of the firm [14]. Based on the available studies, it is evident that there is a lack of research on the organizational performance and dynamic capabilities of MFIs in low-income countries, which in turn leads to a lack of awareness of the importance of organizational performance determinants in low-income countries [16, 19, 20]. In addition, this study focused exclusively on the performance of microfinance institutions (PMFi) in developing countries such as Indonesia and assessed the mediating effect of dynamic capability (DC) on the relationships of TQM, CI, and HRP with PMFi. This study presents novel findings vis-à-vis enhancing the capabilities of MFIs with respect to the RBV. The obtained results regarding the significance of human capabilities, quality management, and customer intelligence in enhancing performance and financial sustainability would benefit various MFIs, particularly in Indonesia.

The remainder of this paper is organized as follows: the next section presents a literature review that discusses the impact of MFIs’ TQM, CI, HRP, and dynamic capabilities on their performance, as well as the mediating role of dynamic capabilities. The research methodology used in this study and the results of the data analysis are presented in the following section. Thereafter, the findings are discussed, leading to conclusions and theoretical and practical implications. Finally, this paper explains and discusses the limitations of the study and suggests areas for further research.

## Literature review

The RBV offers significant theoretical views for organizations to consider in their efforts to intensify their commitment to enhance human resource strategies [21]. According to the RBV, organizations gain competitive advantages and better performance through HRP, which creates unique and valuable human-based resources [22]. The availability of strategic resources that cannot be replicated or substituted helps organizations to acquire and sustain competitive advantage [23]. Burvill et al. [24] highlighted the RBV as a key theory for elucidating the relationship of human resource strategy and practice in a business environment. Innovative organizational strategies through intellectual capability, professional training and experience, and strategic insights of a competent workforce greatly benefit organizations [23].

Evidently, the RBV provides the needed foundation for organizations to consider the development and expansion of organizational practices, which can strengthen and maintain their current business standing and establish a competitive position through strategic resource configuration to gain long-term competitive advantages [25]. Furthermore, the RBV promotes the optimization of strategic resources and measures to capture market opportunities for improved performance [26]. The RBV also postulates the important contributions of internal resources for organizations to gain the required competitive advantage. The RBV highlights the positive influence of human capabilities on organizational performance and competitive advantage [27]. Accordingly, this study assesses the relationships of TQM, CI, and HRP with PMFi, as well as the mediating effect of DC on these hypothesized relationships.

### Hypothesis development

Yunis et al. [28] describe TQM as the holistic procurement and acquisition of management processes for improved dynamic resources and continuous long-term success. It serves as an important approach for organizations to achieve high efficiency, effectiveness, and performance [29]. Numerous organizations have taken an array of steps to adapt and implement TQM to meet various challenges in such dynamic markets. Through TQM, organizations enhance their performance and competitive advantage by leveraging marketing, innovation, and process improvement capabilities [30]. Small and medium-sized enterprises require a quality-oriented organizational culture as well as “hard” and “soft” TQM components to attain sustainable competitive advantages [31].

As a strategic tool, TQM emphasizes on the quality of products and services for competitive advantage. Accordingly, the conceptualization of TQM as a form of strategic resource helps organizations to obtain and disseminate knowledge based on various competencies and distinctive achievements. Focusing on TQM as a source of competitive advantage, Bhaskar [32] empirically demonstrates a significant and positive relationship between TQM and organizational performance. Therefore, this study proposes the following hypotheses:

*H1a*: *TQM has a positive influence on DC*.
*H1b*: *TQM has a positive influence on PMFi*.

It is also crucial for organizations to utilize CI to execute operational strategies for enhanced organizational performance [33, 34]. Through CI, organizations can attract potential customers with high knowledge and skill levels and enhance the knowledge and skill levels of existing customers [35]. Notably, CI reflects the market value and potential market for organizations to achieve high levels of acquisition, satisfaction, loyalty, and retention [36]. An organization’s image and reputation serve as intangible determinants for enhanced organizational performance. In a more recent study, Khalique et al. [37] highlight the strong relationship between CI and organizational performance. Thus, the current study tests the following hypotheses:

*H2a*: *CI has a positive influence on DC*.
*H2b*: *CI has a positive influence on PMFi*.

In addition, the RBV postulates the significance of possessing resources that cannot be replicated and substituted for organizations to achieve competitive advantage, especially in relation to HRP [38]. Dynamic changes in the market drive the implementation of HRP to establish a more strategic and competitive organization [39]. Sabiu et al. [40] highlight the positive relationship between HRP and organizational performance. Theoretically, human resources, in terms of resource innovation, set an organization apart from others [21]. Jackson et al. [41] support this notion and highlight the significant and positive influence of HRP, emphasizing a high level of investment in employees, on organizational performance and competitive advantage. Therefore, the following hypotheses are proposed:

*H3a*: *HRP has a positive influence on DC*.
*H3b*: *HRP has a positive influence on PMFi*.

Lian et al. [42] define DC as the ability of an organization to optimize internal and external resources, such as knowledge, innovation, and systems, with respect to environmental changes. Moreover, DC has been regarded as an important component that influences the performance of an organization [43]. Evolutionary fitness refers to how well DC facilitates an organization’s creation and modification of its basic resources [44]. Generally, DC can be measured in terms of adaptive, absorptive, and innovative capabilities [45]. Absorptive capability refers to the ability to review and gain knowledge from business partners and then apply it for innovation [46]. Integrating capability represents the combination of individual and organizational capabilities to create a new opportunity for organizational success. Meanwhile, the coordination of firm-specific capabilities assigns tasks, resources, and activities to new operational strategies. Consequently, the following hypothesis is presented:

*H4*: *DC has a positive influence on PMFi*.

Meanwhile, Hsu and Wang [47] note the strong influence of the accumulation of DC in terms of intellectual capability and structural capital on organizational performance. Through capabilities, resources can be properly utilized for higher organizational performance [47]. In other words, capabilities serve as key resources for organizations to improve their performance in a dynamic market. An organization with DC can integrate and apply new resources in terms of knowledge and technology, thereby ensuring higher organizational performance [48]. Xing et al. [49] demonstrate the relationship between environmental regulation and organizational performance via DC. Thus, the current study tested the following hypothesis:

*H5*: *DC mediates the influence of TQM*, *CI*, *and HRP on PMFi*.

**Fig 1:**
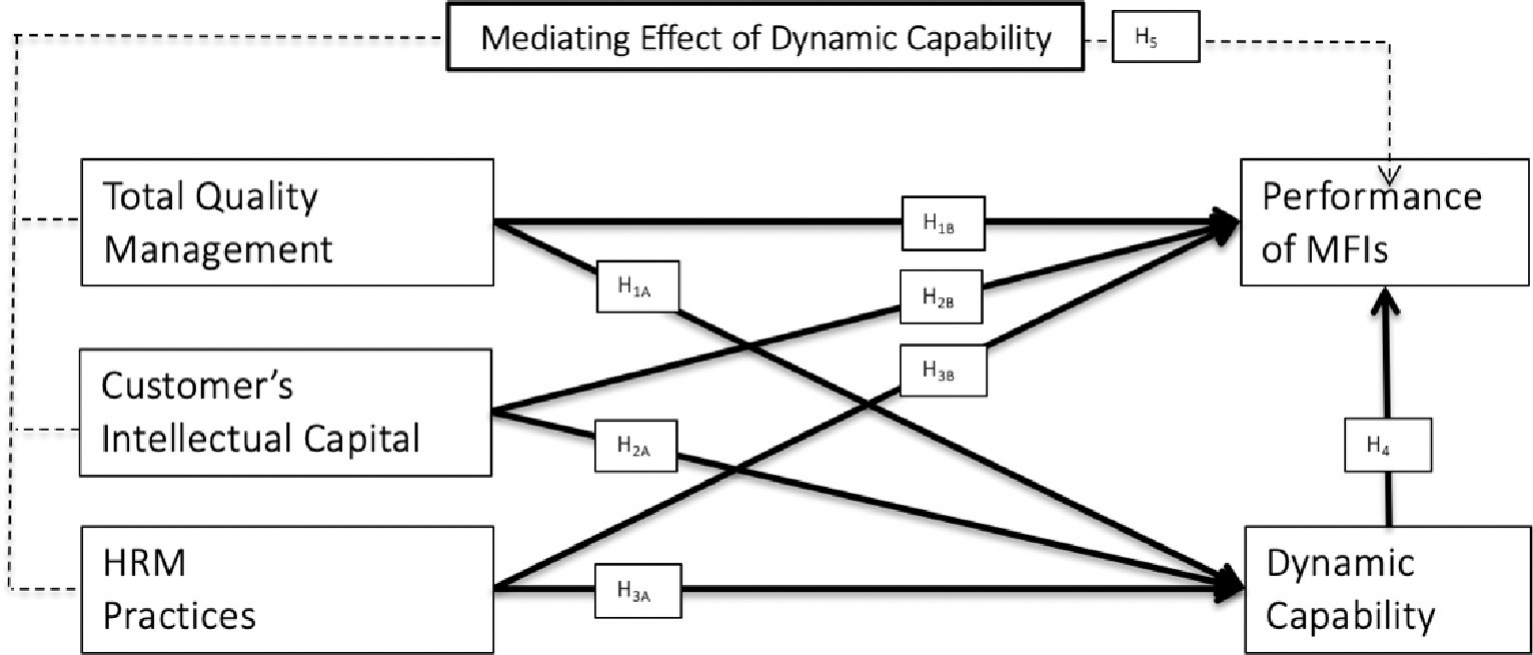
Research framework.

All the associations hypothesized above are presented in Fig 1 below.

## Methodology

This study employs a quantitative cross-sectional approach; an independent questionnaire is used to collect data online. Considering that this study explores information and content that only the management level can access, such as TQM, HRP, and organizational performance, the target population of this study is identified as managers of microfinance companies. Therefore, the target population is managers of MFIs in Indonesia, and these respondents are from an association of credit unions (Induk Koperasi Kredit, INKOPDIT) in West Kalimantan, Indonesia. Owing to the specificity of this study and to protect the anonymity of the respondents’ personal information, all questionnaires were distributed and collected via email and social networking software (WhatsApp). Moreover, all respondents voluntarily participated in the survey and were informed of the general content and purpose of the questionnaire, thereby ensuring the right to information as well as the security and confidentiality of personal information for all the respondents. The research ethics committee of Universitas Widya Dharma Pontianak, Indonesia, approved this study (reference number: 066/RST-FEB/LPPM/IV). Written informed consent was obtained from respondents who participated in the survey.

For this study, the minimum sample size required was determined to be 108 respondents using G-Power 3.1. Thereafter, this study identified 185 potential respondents through a pre-background survey, all from MFIs in West Kalimantan, Indonesia, with more than 1,000 active members and annual revenues of more than Rp 1 billion. Thus, after identifying the potential respondents, purposive sampling was used to distribute a Google questionnaire link and a cover letter to 185 managers (potential respondents) of MFIs in West Kalimantan, Indonesia; 120 complete and valid questionnaire responses were finally obtained.

### Measures of constructs

All measures were adopted from previously validated instruments. Seven items were adopted from Chenhall [50] to measure TQM. In addition, CI in this study was measured using seven items adopted from Bontis [51]. To measure HRP, this study adopted seven items from Lee et al. [52], as well as six items from Bierly and Chakrabarti [53] and Danneels [54] to measure DC. Finally, PMFi was measured in terms of financial and non-financial dimensions such as competitiveness, human capabilities, customer service quality, organizational flexibility, and resource utilization. This study adopted 12 items from Atkinson et al. [55] to measure PMFi. The respondents were required to provide their responses based on a five-point Likert scale, with endpoints being “strongly disagree” (1) and “strongly agree” (5).

### Common Method Bias (CMB)

Harman’s single factor test was used to examine any potential CMB issues [56]. The obtained results revealed five factors. The first factor explained approximately 46.87% of the inconsistency (<50%), hence suggesting the absence of CMB issues in this study. According to Kock [57], the values of the variance inflation factor (VIF) in the full collinearity test that are equal to or lower than 5 indicate that the model does not encounter CMB issues. In the current study, the VIF values for TQM (2.710), CI (2.648), HRP (1.894), DC (2,203), and PMFi (3.235) did not exceed 3.3, thereby confirming the absence of CMB issues.

### Multivariate normality

This study used the Web Power tool to examine the multivariate normality of the data. Accordingly, *p*-values lower than 0.05 indicate the presence of multivariate non-normality in the data. Partial least squares structural equation modeling (PLS-SEM) is recommended for data with multivariate non-normality issues [58].

## Data analysis

### Demographic profile of respondents

Table 1 presents the demographic profile of the respondents. Majority of the respondents in this study were male (64.2%). Furthermore, most of the respondents were married (87.5%), followed by respondents with either a single status (11.7%) or divorced status (0.8%). In addition, approximately 50.0% of the respondents were aged between 34 and 43 years. Regarding the level of education, 73.3% of the respondents reported having a bachelor’s degree, followed by those with a high school degree (15.0%), a diploma degree (8.3%), and a master’s degree (3.3%). The results also revealed that most respondents (70.8%) resided in urban areas, followed by those in rural areas (27.5%). The remaining respondents resided in a district area (1.7%).

**Table 1:** Demographic profile of the respondents.

	n	%		n	%		n	%
*Gender*			*Education*			*Age*		
Male	77	64.2	High school	18	15	17–25 years old	1	0.8
Female	43	35.8	Diploma	10	8.3	26–34 years old	34	28.3
Total	120	100	Bachelor	88	73.3	35–43 years old	60	50
			Master	4	3.3	44–55 years old	24	20
*Marital Status*			Total	120	100	> 55 years old	1	0.8
Married	105	87.5				Total	120	100
Single	14	11.7	*Living Area*					
Divorce	1	0.8	Urban	85	70.8			
Total	120	100	District	2	1.7			
			Rural	33	27.5			
			Total	120	100			

### Reliability and validity

To analyze the external measurement model in depth, this study first validated the reliability and validity of the questionnaires used. The Cronbach’s alpha and Dijkstra–Henseler’s rho were used to ensure the validity of the study questionnaire and the consistency of the measurement scale structure, and the detailed results are presented in Table 2. The recorded values of Cronbach’s alpha and composite reliability for all constructs were above 0.8, exceeding the threshold of 0.7 and thus indicating that the study’s measurement scale had strong reliability and consistency [58]. Furthermore, to understand the validity of the measurement scales in this study in more detail, the data were analyzed through convergent and discriminant validity, where the former was basically determined through average variance extraction (AVE) and factor loading. Based on the results of the data in Table 2, it is evident that the recorded AVE values are above the threshold of 0.5, indicating that the convergent validity of all the measures in this study is reliable and acceptable [58].

**Table 2:** Reliability and validity.

Variables	No. Items	Mean	Standard Deviation	Cronbach’s Alpha	Dijkstra–Henseler’s *rho*	Composite Reliability	Average Variance Extracted
TQM	4	4.060	0.521	0.829	0.836	0.886	0.660
CI	5	4.173	0.471	0.869	0.869	0.905	0.657
HRP	5	4,048	0.496	0.861	0.863	0.900	0.644
DC	5	4.086	0.487	0.888	0.888	0.918	0.694
PMFi	8	4.065	0.463	0.916	0.921	0.931	0.630

**Note:** TQM: Total quality management; CI: Customer intellectual capital; HRP: HRM practices; DC: Dynamic capability; PMFi: Performance of MFIs

The discriminant validity of the constructs was measured based on the Fornell–Larcker criterion [59]and cross-loadings [60]. The obtained results of the criterion are presented in Table 3, while the results of the cross-loadings are provided in **S1 Data**. The results confirmed adequate discriminant validity [61]. According to the results based on the Fornell-Larcker criterion in Table 3, the square root value of the AVE of each latent variable (the diagonal values) exceeded the square root of the other items [59].

**Table 3:** Discriminant validity.

	TQM	CI	DC	HRP	PMFi
Total Quality Management	0.813				
Customer’s Intellectual Capital	0.748	0.811			
Dynamic Capability	0.679	0.639	0.833		
HRM Practices	0.582	0.623	0.614	0.802	
Performance of MFIs	0.693	0.738	0.738	0.653	0.794

**Note:** TQM: Total quality management; CI: Customer intellectual capital; HRP: HRM practices; DC: Dynamic capability; PMFi: Performance of MFIs

### Path analysis

To assess the predictive power of the study model, the structural model was evaluated using predictive correlation (*Q*^*2*^), coefficient of determination (*R*^*2*^), and effect size (*f*^*2*^). Referring to Table 4, the *Q*^*2*^ values for both DC (0.356) and PMFi (0.424) were greater than zero, indicating the presence of predictive power in this study’s framework [62]. As suggested by Hair et al. [63], this study discriminated the explanatory power of its model using *R*^*2*^; the larger the *R*^*2*^ value, the stronger the explanatory power. The *R*^*2*^ values of DC (0.546) and PMFi (0.711) in this study ranged from 0.5 to 0.75, indicating that the explanatory power of the model was moderate and significant. In addition, this study assessed the effect sizes of the predictor variables using Hair et al.’s [57]research threshold interpretation of *f*^*2*^; where *f*^*2*^ values of 0.005, 0.01, and 0.025 were considered to be small, medium, and large effect sizes, respectively. Following this criterion, among the factors affecting PMFI in this study, the *f*^*2*^ value for TQM (0.010) was considered to have a medium effect size; CI (0.127), HRP (0.042), and DC (0.272) had *f*^*2*^ values greater than 0.025 and were considered to have large effect sizes. Among the factors affecting DC, TQM (0.136), CI (0.027), and HRP (0.101) all had relatively large effect sizes.

**Table 4:** Path coefficients.

Hypothesis		Beta	*t*	*p*	*R* ^2^	*f* ^*2*^	Q^2^	Decision
*Factors Effecting DC *								
H_1a_	TQM ➜ DC	0.383	2.940	0.003	0.546	0.136	0.356	Supported
H_2a_	CI ➜ DC	0.178	1.475	0.141		0.027		Rejected
H_3a_	HRP ➜ DC	0.280	2.830	0.000		0.101		Supported
*Factor Effecting PMFI*								
H_1b_	TQM ➜ PMFi	0.089	0.868	0.386	0.711	0.010	0.424	Rejected
H_2b_	CI ➜ PMFi	0.311	3.609	0.000		0.127		Supported
H_3b_	HRP ➜ PMFi	0.152	1.430	0.153		0.042		Rejected
H_4_	DC ➜ PMFi	0.416	4.765	0.000		0.272		Supported

**Note:** TQM: Total quality management; CI: Customer intellectual capital; HRP: HRM practices; DC: Dynamic capability; PMFi: Performance of MFIs

Table 4 presents the results of the path analysis. The results revealed a statistically significant and positive influence of TQM (*β* = 0.383, *p* = 0.003) and HRP (*β* = 0.280, *p* = 0.000) on DC. Second, the influence of CI on DC was positive but statistically insignificant (*β* = 0.178, *p* = 0.141). Third, the results demonstrated a positive but statistically insignificant influence of TQM (*β* = 0.089, *p* = 0.386) and HRP (*β* = 0.152, *p* = 0.153) on PMFi. However, the influence of CI (*β* = 0.311, *p* = 0.000) on PMFi was statistically significant and positive. Last but not least, the results revealed a statistically significant and positive influence of DC on PMFi (*β* = 0.416, *p* = 0.000).

### Mediating effect

This study hypothesized a mediating effect of DC on the relationships of TQM, CI, and HRP with PMFi. Referring to the results in Table 5, the influence of TQM on PMFi recorded a positive coefficient value of 0.16 (*p* = 0.025), thereby suggesting a statistically significant indirect relationship. In other words, DC mediated the relationship between TQM and PMFi. Meanwhile, the influence of CI on PMFi had a positive coefficient value of 0.074 (*p* = 0.148), implying a statistically insignificant relationship. Thus, DC did not mediate the relationship between CI and PMFi. Finally, the influence of HRP on PMFi had a positive coefficient value of 0.117 (*p* = 0.017), indicating a statistically significant indirect relationship. Thus, DC did mediate the relationship between HRP and PMFi.

**Table 5:** Mediating effect.

Associations	Beta	*t*	*p*	Decision
TQM ➜ DC ➜ PMFi	0.160	2.251	0.025	Supported
CI ➜ DC ➜ PMFi	0.074	1.448	0.148	Rejected
HRP ➜ DC ➜ PMFi	0.117	2.395	0.017	Supported

**Note:** TQM: Total quality management; CI: Customer intellectual capital; HRP: HRM practices; DC: Dynamic capability; PMFi: Performance of MFIs

## Discussion

With respect to the RBV, this study quantitatively examined the mediating effect of DC on the relationships of TQM, CI, and HRP with PMFi from the perspective of purposively selected managers of MFIs in Indonesia. As an extension of the RBV, DC reflects the capacity of MFIs to integrate, build, and reconfigure their internal and external resources [64]. Notably, MFIs with higher levels of DC can enhance their innovation plans in time and develop valuable capabilities that promote a higher level of organizational performance. Overall, the obtained PLS-SEM results demonstrated the importance of DC in relation to TQM, CI, and HRP for PMFi.

Based on the results, TQM has a critical impact on the dynamic capabilities of MFIs but has an insignificant effect on organizational performance; thus, H1_a_ is supported and H1_b_ is rejected. Furthermore, TQM is a strong predictor of DC, which is consistent with the findings of Shuaib et al. [65] in their study on dynamic capabilities and quality management, as well as with those of Sahoo [66] in India who explores quality management, innovation capability, and firm performance. This finding is consistent with those of Silva et al. [30] in that organizations take steps to adapt and implement TQM, which includes strategies such as marketing, innovation, and process improvement capabilities to improve and enhance their dynamic capabilities and thus gain a competitive advantage to meet the various challenges of this dynamic market. However, the direct relationship is indeed surprising, as the relationship between TQM and organizational performance (PMFi) is not statistically significant, that is, the evidence obtained in this study is insufficient to support the relationship between TQM and PMFi, which is contrariwise to Bhaskar’s [32] findings. Ong and Tan’s [67]study on manufacturing organizations in the electronics industry in Malaysia found no significant direct relationship between TQM and organizational performance, but a direct relationship could be generated with the mediation of knowledge management, similar to the findings of this study that TQM could generate a significant direct relationship with organizational performance (PMFi) through the mediation of dynamic organizational capabilities. This result may be related to factors such as dynamic markets, which may prioritize responding to external market changes and maintaining their own competitiveness, thus paying less attention to internal organizational factors (e.g., TQM and HRP) over time [68].

Second, by addressing the relationship between CI, DC, and PMFi, the results of this study confirm that CI has an insignificant effect on DC but is a strong predictor of PMFi, which leads to H2_a_ being rejected and H2_b_ being supported. In a study of the hospitality industry, Elsharnouby and Elbanna [69] find that human capital, directly and indirectly through marketing-related dynamic capabilities, plays a key role in developing competitive advantage but cannot directly influence the dynamic capabilities of the firm, which is consistent with the results of H2a in this study. In other words, organizations should consider their customers’ needs for high quality and value and use their capabilities to deliver a correspondingly high-quality product or service [69, 70]. Interestingly, this study found adequate empirical evidence to support H2_b_ regarding the relationship between CI and PMFi, which supported the current literature on how organizations that emphasize CI could achieve higher performance [37, 71]. To achieve higher organizational performance, MFIs should refrain from compromising their service quality for customers, demonstrate their concern for customer feedback, and sustain their relationships with customers to promote customer trust.

Additionally, this study acquired adequate empirical evidence to support H3_a_ regarding the relationship between HRP and DC, thus supporting the findings of Dahie and Mohamed [39]. The previous study highlighted how HRP could help organizations to form the required competence, adaptability, and intelligence to sustain their performance in this highly competitive and changing environment. Unexpectedly, HRP was found to have an insignificant influence on PMFi (H3_b_), thereby contradicting the results reported by Jackson et al. [41] and Amjad et al. [72]. This may be because of the different sociocultural contexts involved. Contrary to the findings of this study, Amjad et al. [72] found that HRP continued to influence organizational and employee performances in terms of organizational sustainability. The previous study demonstrated the significant and positive influence of HRP, focusing on a high level of investment in employees, organizational performance, and competitive advantage. Meanwhile, DC was found to have significant and positive influence on PMFi (H4) in this study. Chien and Tsai [48], Drnevich and Kriauciunas [73] reported similar findings on how improving organizational capabilities could improve organizational profitability and performance. This finding was revalidated in recent studies by Loureiro et al. [74], Nguyen et al. [75], and Permatasari et al. [76]in organizations in the craft, tourism, and healthcare sectors, which was consistent with the results of the present study.

Regarding the indirect relationships of TQM, CI, and HRP with PMFi, this study finds empirical evidence that partially supported H5. First, this study establishes a significant mediating effect of DC on the relationship between TQM and PMFi. Roberts and Grover [77] report similar findings regarding the positive influence of DC on the role of TQM, with extensive and formal training for employees to promote small-group interactions. Second, this study reports an insignificant mediating effect of DC on the relationship between CI and PMFi. Third, this study finds a significant mediating effect of DC on the relationship between HRP and PMFi. According to the RBV, the implementation of DC enhances PMFi in terms of profitability, market standing, and competitive advantage [64]. Based on these findings, it is evident that MFIs need to consider the roles of TQM, CI, HRP, and DC in boosting and sustaining their performance, particularly in developing countries. This study demonstrates the importance of DC for organizations to attain competitiveness and adaptability in this rapidly changing environment. Furthermore, this study successfully demonstrates the role of the RBV in the sustainable performance of MFIs, particularly regarding the mediating effect of DC in enhancing the influence of TQM and HRP on PMFi.

## Conclusions

The RBV served as the current study’s underlying theoretical basis for assessing the mediating effect of DC on the relationships of TQM, CI, and HRP with PMFi. Based on the results obtained, only CI demonstrated a significant and positive direct influence on PMFi. In addition, this study demonstrated a positive mediating effect of DC on the relationships of TQM and HRP with PMFi. This study revealed the close relationships of the RBV with quality management, including tangible and intangible assets that are difficult to replicate or substitute. Evidently, MFIs should make use of their unique resources and qualities of their employees under effective organizational management, leadership, and control to improve and sustain their performance. Furthermore, as part of the RBV, MFIs should consider TQM, CI, and HRP to ensure high-quality outputs.

This study has significant theoretical implications for MFIs. It also offers valuable insights into factors that significantly influence PMFi in developing countries. Additionally, this study presents empirical evidence in connection to the influence of TQM, CI, HRP, and DC on PMFi, particularly within the context of emerging economies. Moreover, this study empirically proved the relevance of the RBV in elucidating the relationships of TQM, CI, HRP, and DC with PMFi from the viewpoint of Indonesian MFI managers.

This study presents valuable insights that would benefit MFIs and policymakers, particularly regarding how organizations can use TQM, CI, and HRP to enhance their performance. The findings of this study would encourage MFIs to attain enhanced performance and competitive advantages by focusing on TQM, CI, HRP, and DC. This study empirically demonstrated the mediating role of DC in boosting the influence of TQM and HRP on PMFi. Evidently, quality management would deliver high-quality training and managerial leadership to motivate effective interactions among employees, thus contributing to a higher level of innovative performance. Through HRP, MFIs can establish a positive organizational culture that boosts employees’ relationships and capabilities under effective managerial leadership. Likewise, MFIs should make efforts to strengthen their dynamic sensing, learning, integration, and coordination skills for enhanced performance. Moreover, this study offers a unique perspective on the mediating role of DC in boosting the relationship between TQM and PMFi, particularly for MFIs in developing countries such as Indonesia.

Notwithstanding the significant contributions of this study in terms of theoretical and practical applications, some limitations and restrictions remain. First, this study adopts a cross-sectional research design and quantitative approach that lacks the ability to test for causal relationships, which in turn limits the generalizability of the findings. This study neither implies nor examines the existence of causal relationships between factors such as an organization’s TQM, DC, and PMFi. Second, this study focuses on small credit firms, which are the type of institutions that examine organizational performance through financial performance, but does not give much consideration to other potential financial determinants. Third, the present study only examines microfinance firms in selected regions of Indonesia, which leads to a limited generalization of the findings, and may be inapplicable in regional and national contexts and firm situations.

The above-stated reasons lead to several limitations in the findings of this study, which need to be addressed and amended in future studies. First, future researchers should consider a longitudinal research design and a mixed methods approach to explore the causal relationship between various potential determinants and strategic objectives (e.g., the concept of innovation). Second, when conducting further research on credit institutions and banking firms, financial determinants and measures ought to be considered and added to comprehensively examine the performance of this category of firms and organizations. Broad-based indicators will produce more reliable results. In addition, future studies should consider expanding the sample and geographical scope so that a broader and deeper perspective can be taken to explain the organizational performance, social objectives, market competitiveness, and sustainability of MFIs.

## Supporting information

S1 Data (CSV)

## References

[1] Gatto A. (2018). Historical roots of microcredit and usury: The role ofmonti di pietàin Italy and in the Kingdom of Naples in XV-xx centuries. Journal of International Development, 30(5), 911–914. 10.1002/jid.3386

[2] Gupta P. K., & Sharma S. (2021). Literature review on effect of microfinance institutions on poverty in South Asian countries and their sustainability. International Journal of Emerging Markets. 10.1108/ijoem-07-2020-0861

[3] Li L. Y., Hermes N., & Meesters A. (2019). Convergence of the performance of Microfinance Institutions: A Decomposition Analysis. Economic Modelling, 81, 308–324. 10.1016/j.econmod.2019.05.014

[4] Milana C., & Ashta A. (2020). Microfinance and financial inclusion: Challenges and opportunities. Strategic Change, 29(3), 257–266. 10.1002/jsc.2339

[5] Gatto A., & Sadik-Zada E. R. (2022). Access to microfinance as a resilience policy to address Sustainable Development Goals: A content analysis. Heliyon, 8(10). 10.1016/j.heliyon.2022.e10860 36267370 PMC9578961

[6] Hermes N., & Hudon M. (2018). Determinants of the performance of microfinance institutions: A systematic review. Journal of Economic Surveys, 32(5), 1483–1513. 10.1111/joes.12290

[7] Teece D. J. (2012). Dynamic capabilities: Routines versus entrepreneurial action. Journal of Management Studies, 49(8), 1395–1401. 10.1111/j.1467-6486.2012.01080.x

[8] Joseph O. O., & Kibera F. (2019). Organizational culture and performance: Evidence from microfinance institutions in Kenya. SAGE Open, 9(1), 215824401983593. 10.1177/2158244019835934

[9] Lacalle-Calderon M., Perez-Trujillo M., & Neira I. (2018). Does microfinance reduce poverty among the poorest? A macro quantile regression approach. The Developing Economies, 56(1), 51–65. 10.1111/deve.12159

[10] Bharti N., & Malik S. (2021). Financial inclusion and the performance of microfinance institutions: Does social performance affect the efficiency of microfinance institutions? Social Responsibility Journal, 18(4), 858–874. 10.1108/srj-03-2020-0100

[11] UNHCR. (2022). Indonesia fact Sheet–February 2022-final–unhcr.org. statistical report. Retrieved April 11, 2023, from https://www.unhcr.org/id/wp-content/uploads/sites/42/2022/04/Indonesia-Fact-Sheet-February-2022-FINAL.pdf

[12] Iqbal S., Nawaz A., & Ehsan S. (2019). Financial performance and corporate governance in microfinance: Evidence from Asia. Journal of Asian Economics, 60, 1–13. 10.1016/j.asieco.2018.10.002

[13] Kusuma S. E., Sumarwan A., & Kusumajati T. O. (2022). The role of Integrative Approach for Enhancing Credit Union Sustainability: A reflection on the Indonesian Credit Union Movement. Jurnal Ekonomi Pembangunan: Kajian Masalah Ekonomi Dan Pembangunan, 23(1), 31–42. 10.23917/jep.v23i1.17851

[14] Akbar T., & Siti-Nabiha A. K. (2021). Objectives and measures of performance of Islamic microfinance banks in Indonesia: The stakeholders’ Perspectives. ISRA International Journal of Islamic Finance, 14(2), 124–140. 10.1108/ijif-11-2020-0231

[15] Wernerfelt B. (1984). A resource-based view of the firm. Strategic Management Journal, 5(2), 171–180. 10.1002/smj.4250050207

[16] Muithya V., & Muathe S. (2020). Dynamic capabilities and performance in the context of microfinance institutions in Kenya: An exploratory study. Journal of Business, Economics and Management Works, 7(08), 15–29.

[17] Usrof H. J. H., & Elmorsey R. M. (2016). Relationship between HRM and TQM and its influence on organizational sustainability. International Journal of Academic Research in Accounting, Finance and Management Sciences, 6(2). 10.6007/ijarafms/v6-i2/2036

[18] Al-Tal M. J., & Emeagwali O. L. (2019). Knowledge-based HR practices and innovation in smes. Organizacija, 52(1), 6–21. 10.2478/orga-2019-0002

[19] Magistretti S., Pham C. T., & Dell’Era C. (2021). Enlightening the dynamic capabilities of Design Thinking in fostering digital transformation. Industrial Marketing Management, 97, 59–70. 10.1016/j.indmarman.2021.06.014

[20] Bhaduri S., & Selarka E. (2022). The zombie story: Credit boom and the rise of zombie firms in the Indian economy. Maladies of the Indian Banking Sector, 160–183. 10.1017/9781009225472.009

[21] Collins C. J. (2020). Expanding the resource based view model of Strategic Human Resource Management. The International Journal of Human Resource Management, 32(2), 331–358. 10.1080/09585192.2019.1711442

[22] Hee O. C., & Shanmugam N. (2019). A review of Human Resource Change Management Strategies in the Digital Era. International Journal of Academic Research in Business and Social Sciences, 9(3). 10.6007/ijarbss/v9-i3/5713

[23] Barney J. B. (2001). Resource-based theories of competitive advantage: A ten-year retrospective on the resource-based view. Journal of Management, 27(6), 643–650. 10.1177/014920630102700602

[24] Burvill S. M., Jones-Evans D., & Rowlands H. (2018). Reconceptualising the principles of Penrose’s (1959) theory and the resource based view of the firm. Journal of Small Business and Enterprise Development, 25(6), 930–959. 10.1108/jsbed-11-2017-0361

[25] Eisenhardt K. M., & Martin J. A. (2017). Dynamic capabilities: What are they? The SMS Blackwell Handbook of Organizational Capabilities, 341–363. 10.1002/9781405164054.ch21

[26] Pitelis C. (2009). Edith Penrose’s ‘the theory of the growth of the firm’ fifty years later. SSRN Electronic Journal. 10.2139/ssrn.1477885

[27] Colbert B. A. (2004). The complex resource-based view: Implications for theory and practice in strategic human resource management. The Academy of Management Review, 29(3), 341. 10.2307/20159047

[28] Yunis M., Jung J., & Chen S. (2013). TQM, strategy, and performance: A Firm‐level analysis. International Journal of Quality & Reliability Management, 30(6), 690–714. 10.1108/02656711311325638

[29] Sweis R., Ismaeil A., Obeidat B., & Kanaan R. K. (2019). Reviewing the literature on Total Quality Management and organizational performance. Journal of Business & Management (COES&RJ-JBM), 7(3), 192–215. 10.25255/jbm.2019.7.3.192.215

[30] Silva G. M., Gomes J., P., Lages L. F., & Pereira Z. (2014). The role of TQM in strategic product innovation: An empirical assessment. International Journal of Operations & Production Management, 34(10), 1307–1337. 10.1108/ijopm-03-2012-0098

[31] Sutrisno T. F. (2019). Relationship between total quality management element, operational performance and organizational performance in food production smes. JURNAL APLIKASI MANAJEMEN, 17(2), 285–294. 10.21776/ub.jam.2019.017.02.11

[32] Bhaskar H. L. (2020). Establishing a link among total quality management, market orientation and organizational performance. The TQM Journal, 32(6), 1507–1524. 10.1108/tqm-01-2019-0012

[33] Abualoush S., Masa’deh R., Bataineh K., & Alrowwad A. (2018). The role of Knowledge Management Process and intellectual capital as intermediary variables between knowledge management infrastructure and organization performance. Interdisciplinary Journal of Information, Knowledge, and Management, 13, 279–309. 10.28945/4088

[34] Ur Rehman S., Elrehail H., Alsaad A., & Bhatti A. (2021). Intellectual Capital and innovative performance: A mediation-moderation perspective. Journal of Intellectual Capital, 23(5), 998–1024. 10.1108/jic-04-2020-0109

[35] Odhon’g E. A., & Omolo J. (2015). Effect of human capital investment on organizational performance of pharmaceutical companies in Kenya. Global Journal of Human Resource Management, 3(6), 1–29.

[36] Hariyati H., Tjahjadi B., & Soewarno N. (2019). The mediating effect of Intellectual Capital, Management Accounting Information Systems, internal process performance, and Customer Performance. International Journal of Productivity and Performance Management, 68(7), 1250–1271. 10.1108/ijppm-02-2018-0049

[37] Khalique M., Bontis N., Shaari J. A., Yaacob M. R., & Ngah R. (2018). Intellectual capital and organizational performance in Malaysian knowledge-intensive smes. International Journal of Learning and Intellectual Capital, 15(1), 20. 10.1504/ijlic.2018.088345

[38] Nyberg A. J., & Wright P. M. (2015). 50 years of Human Capital Research: Assessing what we know, Exploring where we go. Academy of Management Perspectives, 29(3), 287–295. 10.5465/amp.2014.0113

[39] Dahie A. M., & Mohamed R. A. (2017). Human Resource Management Practice and organizational performance: Case study from hormuud telecom in Mogadishu-somalia. European Researcher, 8(2). 10.13187/er.2017.2.78

[40] Sabiu M. S., Ringim K. J., Mei T. S., & Joarder M. H. (2019). Relationship between human resource management practices, ethical climates and organizational performance, the missing link. PSU Research Review, 3(1), 50–69. 10.1108/prr-12-2016-0022

[41] Jackson S. E., Schuler R. S., & Jiang K. (2014). An aspirational framework for Strategic Human Resource Management. Academy of Management Annals, 8(1), 1–56. 10.5465/19416520.2014.872335

[42] Lian Z., Liu Q., & Guo Y. (2020). Research on antecedent conditions of TQM and technological innovation process coupling from configuration perspective. Accounting and Corporate Management, 2(1), 31–41. 10.23977/acccm.2020.020103

[43] Laaksonen O., & Peltoniemi M. (2016). The essence of dynamic capabilities and their measurement. International Journal of Management Reviews, 20(2), 184–205. 10.1111/ijmr.12122

[44] Ferreira J., & Coelho A. (2020). Dynamic capabilities, innovation and branding capabilities and their impact on competitive advantage and SME’s performance in Portugal: The moderating effects of entrepreneurial orientation. International Journal of Innovation Science, 12(3), 255–286. 10.1108/ijis-10-2018-0108

[45] Wang C. L., & Ahmed P. K. (2007). Dynamic capabilities: A review and research agenda. International Journal of Management Reviews, 9(1), 31–51. 10.1111/j.1468-2370.2007.00201.x

[46] Liu H. Y., & Hsu C. W. (2011). Antecedents and consequences of corporate diversification. Management Decision, 49(9), 1510–1534. 10.1108/00251741111173961

[47] Hsu L.-C., & Wang C.-H. (2010). Clarifying the effect of intellectual capital on performance: The mediating role of Dynamic capability. British Journal of Management, 23(2), 179–205. 10.1111/j.1467-8551.2010.00718.x

[48] Chien S. Y., & Tsai C. H. (2012). Dynamic Capability, knowledge, learning, and firm performance. Journal of Organizational Change Management, 25(3), 434–444. 10.1108/09534811211228148

[49] Xing X., Liu T., Shen L., & Wang J. (2020). Linking Environmental Regulation and financial performance: The mediating role of Green Dynamic Capability and Sustainable Innovation. Sustainability, 12(3), 1007. 10.3390/su12031007

[50] Chenhall R. H. (1997). Reliance on manufacturing performance measures, Total Quality Management and organizational performance. Management Accounting Research, 8(2), 187–206. 10.1006/mare.1996.0038

[51] Bontis N. (1998). Intellectual capital: An exploratory study that develops measures and models. Management Decision, 36(2), 63–76. 10.1108/00251749810204142

[52] Lee F.-H., Lee T.-Z., & Wu W.-Y. (2010). The relationship between Human Resource Management Practices, business strategy and firm performance: Evidence from steel industry in Taiwan. The International Journal of Human Resource Management, 21(9), 1351–1372. 10.1080/09585192.2010.488428

[53] Bierly P. E., & Chakrabarti A. K. (1996). Technological Learning, strategic flexibility, and new product development in the pharmaceutical industry. IEEE Transactions on Engineering Management, 43(4), 368–380. 10.1109/17.543979

[54] Danneels E. (2002). The dynamics of product innovation and firm competences. Strategic Management Journal, 23(12), 1095–1121. 10.1002/smj.275

[55] Atkinson A., & Epstein M., Atkinson A., and Epstein M. (2000). Measure for measure: Realizing the power of the balanced scorecard. CMA Management, 74(7), 22–28.

[56] Podsakoff P. M., MacKenzie S. B., Lee J.-Y., & Podsakoff N. P. (2003). Common method biases in behavioral research: A critical review of the literature and recommended remedies. Journal of Applied Psychology, 88(5), 879–903. 10.1037/0021-9010.88.5.879 14516251

[57] Kock N. (2017). Common method bias: A full Collinearity Assessment Method for PLS-SEM. Partial Least Squares Path Modeling, 245–257. 10.1007/978-3-319-64069-3_11

[58] Hair J. F., M., Ringle H. G. T.,, Sarstedt C. M.,, Danks M.,, N. P., & Ray S. (2021). Partial least squares structural equation modeling (Pls-Sem) using r: A workbook. Springer.

[59] Fornell C., & Larcker D. F. (1981). Evaluating structural equation models with unobservable variables and measurement error. Journal of Marketing Research, 18(1), 39. 10.2307/3151312

[60] Hair J. F., Risher J. J., Sarstedt M., & Ringle C. M. (2019). When to use and how to report the results of PLS-SEM. European Business Review, 31(1), 2–24. 10.1108/ebr-11-2018-0203

[61] Kline E., Wilson C., Ereshefsky S., Tsuji T., Schiffman J., Pitts S., & Reeves G. (2012). Convergent and discriminant validity of attenuated psychosis screening tools. Schizophrenia Research, 134(1), 49–53. 10.1016/j.schres.2011.10.001 22036199

[62] Sarstedt M., Ringle C. M., & Hair J. F. (2017). Partial least squares structural equation modeling. Handbook of Market Research, 1–40. 10.1007/978-3-319-05542-8_15-1

[63] Hair J. F. Jr., Matthews L. M., Matthews R. L., & Sarstedt M. (2017). PLS-SEM or CB-SEM: Updated guidelines on which method to use. International Journal of Multivariate Data Analysis, 1(2), 107. 10.1504/ijmda.2017.10008574

[64] Barney J. B. (1995). Looking inside for competitive advantage. Academy of Management Perspectives, 9(4), 49–61. 10.5465/ame.1995.9512032192

[65] Shuaib K. M., He Z., & Song L. (2021). Effect of organizational culture and Quality Management on innovation among Nigerian manufacturing companies: The mediating role of dynamic capabilities. Quality Management Journal, 28(4), 223–247. 10.1080/10686967.2021.1962773

[66] Sahoo S. (2019). Quality Management, innovation capability and firm performance. The TQM Journal, 31(6), 1003–1027. 10.1108/tqm-04-2019-0092

[67] Ong E. C., & Tan C. L. (2022). Soft TQM, agility, and knowledge management deliver organizational performance: A study of Malaysian manufacturing organizations in the Electrical and Electronics Sector. Global Business and Organizational Excellence, 41(4), 28–47. 10.1002/joe.22155

[68] Pereira V., Mellahi K., Temouri Y., Patnaik S., & Roohanifar M. (2019). Investigating dynamic capabilities, agility and knowledge management within emnes-longitudinal evidence from Europe. Journal of Knowledge Management, 23(9), 1708–1728. 10.1108/jkm-06-2018-0391

[69] Elsharnouby T. H., & Elbanna S. (2021). Change or perish: Examining the role of human capital and dynamic marketing capabilities in the hospitality sector. Tourism Management, 82, 104184. 10.1016/j.tourman.2020.104184

[70] Landroguez S. M., Castro C. B., & Cepeda‐Carrión G. (2011). Creating dynamic capabilities to increase customer value. Management Decision, 49(7), 1141–1159. 10.1108/00251741111151181

[71] Haldorai K., Kim W. G., & Garcia R. L. F. (2022). Top management green commitment and green intellectual capital as enablers of Hotel Environmental Performance: The mediating role of green human resource management. Tourism Management, 88, 104431. 10.1016/j.tourman.2021.104431

[72] Amjad F., Abbas W., Zia-UR-Rehman M., Baig S. A., Hashim M., Khan A., et al. (2021). Effect of green human resource management practices on organizational sustainability: The mediating role of environmental and employee performance. Environmental Science and Pollution Research. doi: 10.1007/s11356-020-11307-9 33527245

[73] Drnevich P. L., & Kriauciunas A. P. (2011). Clarifying the conditions and limits of the contributions of ordinary and dynamic capabilities to relative firm performance. Strategic Management Journal, 32(3), 254–279. 10.1002/smj.882

[74] Loureiro R., Ferreira J. J., & Simões J. (2021). Understanding Healthcare sector organizations from a Dynamic Capabilities Perspective. European Journal of Innovation Management, 26(2), 588–614. 10.1108/ejim-02-2021-0085

[75] Nguyen H. T., Pham H. S., & Freeman S. (2022). Dynamic capabilities in tourism businesses: Antecedents and outcomes. Review of Managerial Science. 10.1007/s11846-022-00567-z

[76] Permatasari A., Dhewanto W., & Dellyana D. (2022). The role of traditional knowledge-based dynamic capabilities to improve the sustainable performance of weaving craft in Indonesia. Journal of Enterprising Communities: People and Places in the Global Economy. 10.1108/jec-11-2021-0156

[77] Roberts N., & Grover V. (2012). Investigating firm’s customer agility and firm performance: The importance of aligning sense and respond capabilities. Journal of Business Research, 65(5), 579–585. 10.1016/j.jbusres.2011.02.009

